# Proteomic Alterations and Novel Markers of Neurotoxic Reactive Astrocytes in Human Induced Pluripotent Stem Cell Models

**DOI:** 10.3389/fnmol.2022.870085

**Published:** 2022-05-03

**Authors:** David Labib, Zhen Wang, Priya Prakash, Matthew Zimmer, Matthew D. Smith, Paul W. Frazel, Lilianne Barbar, Maria L. Sapar, Peter A. Calabresi, Junmin Peng, Shane A. Liddelow, Valentina Fossati

**Affiliations:** ^1^The New York Stem Cell Foundation Research Institute, New York, NY, United States; ^2^Department of Structural Biology, St. Jude Children’s Research Hospital, Memphis, TN, United States; ^3^Department of Developmental Neurobiology, St. Jude Children’s Research Hospital, Memphis, TN, United States; ^4^Neuroscience Institute, NYU Grossman School of Medicine, New York, NY, United States; ^5^Department of Neurology, Johns Hopkins University, Baltimore, MD, United States; ^6^Solomon H. Snyder Department of Neuroscience, Johns Hopkins University, Baltimore, MD, United States; ^7^Center for Proteomics and Metabolomics, St. Jude Children’s Research Hospital, Memphis, TN, United States; ^8^Department of Neuroscience and Physiology, NYU Grossman School of Medicine, New York, NY, United States; ^9^Department of Ophthalmology, NYU Grossman School of Medicine, New York, NY, United States; ^10^Parekh Center for Interdisciplinary Neurology, NYU Grossman School of Medicine, New York, NY, United States

**Keywords:** reactive astrocytes, induced pluripotent stem cells, proteomics, surface markers, inflammation, neurodegenerative diseases

## Abstract

Astrocytes respond to injury, infection, and inflammation in the central nervous system by acquiring reactive states in which they may become dysfunctional and contribute to disease pathology. A sub-state of reactive astrocytes induced by proinflammatory factors TNF, IL-1α, and C1q (“TIC”) has been implicated in many neurodegenerative diseases as a source of neurotoxicity. Here, we used an established human induced pluripotent stem cell (hiPSC) model to investigate the surface marker profile and proteome of TIC-induced reactive astrocytes. We propose VCAM1, BST2, ICOSL, HLA-E, PD-L1, and PDPN as putative, novel markers of this reactive sub-state. We found that several of these markers colocalize with GFAP^+^ cells in post-mortem samples from people with Alzheimer’s disease. Moreover, our whole-cells proteomic analysis of TIC-induced reactive astrocytes identified proteins and related pathways primarily linked to potential engagement with peripheral immune cells. Taken together, our findings will serve as new tools to purify reactive astrocyte subtypes and to further explore their involvement in immune responses associated with injury and disease.

## Introduction

Astrocytes have been increasingly recognized as drivers of pathophysiology in many degenerative diseases of the central nervous system (CNS), such as Alzheimer’s disease (AD) and multiple sclerosis (MS) (Phatnani and Maniatis, 2015; Franklin et al., 2021). Under physiological conditions, astrocytes maintain brain homeostasis through several functions that involve retaining the integrity of the blood-brain barrier, providing metabolic support to neurons, promoting synapse formation, maturation and pruning, and several other integral physiological functions (Liddelow and Barres, 2017; Han et al., 2021). In injury, disease, and aging, astrocytes undergo functional dysregulation marked by morphological and molecular changes, which disrupt their homeostatic functions, and have been collectively characterized as reactive astrocytes. Many heterogeneous reactive sub-states have been described and they range from having detrimental to protective effects on the surrounding CNS cells (Liddelow et al., 2017; Hyvärinen et al., 2019). These heterogeneous sub-states are highly context dependent–varying with time (from the initial insult and perhaps circadian rhythms; Griffin et al., 2019), location (Hasel et al., 2021), and the source of the reactive stimuli, such as inflammation (Zamanian et al., 2012; Liddelow et al., 2017; Wheeler et al., 2020) or aging (Boisvert et al., 2018; Clarke et al., 2018). The heterogeneity of reactive astrocyte responses that may be attributed to these (and potentially other) variables remains understudied. While transcriptomic investigations have provided key insights, a general lack of functional characterization of specific reactive astrocyte sub-states has limited our understanding of their roles in disease, injury, and following infection.

Studies in rodent models have identified and characterized multiple reactive states. One specific reactive sub-state that has been extensively investigated is induced by the proinflammatory factors TNF, IL-1α, and C1q (hereafter termed “TIC”). This sub-state of reactive astrocytes loses many physiological functions and gains a potent neurotoxic capacity in both rodent and human astrocytes (Liddelow et al., 2017; Barbar et al., 2020a; Guttenplan et al., 2021). The neurotoxic capacity of TIC-induced reactive astrocytes has been highlighted both *in vitro* (Liddelow et al., 2017; Barbar et al., 2020a) and *in vivo* in many disease models (Shi et al., 2017; Yun et al., 2018; Gibson et al., 2019; Hartmann et al., 2019; Kleffman et al., 2019; Guttenplan et al., 2020a,b; Lopez-Sanchez et al., 2020; Sterling et al., 2020; Fernández-Castañeda et al., 2022). Their active contribution to human neurodegenerative diseases has also been putatively attributed to their location in regions of neurodegeneration in human post-mortem tissue (Liddelow et al., 2017; Hartmann et al., 2019; Holst et al., 2019; Guttenplan et al., 2020b; King et al., 2020). More recently, the identity of the TIC-induced reactive astrocyte sub-state secreted neurotoxin has been identified. Rather than a protein, a selection of long-chain saturated lipids packaged in lipoparticles drive neuron apoptosis *in vitro* and in acute axonal injury *in vivo* (Guttenplan et al., 2021). Leveraging the versatility of human induced pluripotent stem cell (hiPSC) technologies, we previously reported that hiPSC-derived astrocytes can be highly purified using the surface molecule CD49f (Barbar et al., 2020a). These CD49f^+^ cells resemble mature astrocytes–they express canonical astrocyte markers (e.g., ALDH1L1, S100β, EAAT1) and mimic critical *in vivo* functions including glutamate uptake, phagocytosis, and neuronal maturation support. Moreover, we demonstrated that TIC stimulation induced a reactive state in hiPSC-derived astrocytes that transcriptomically resembles rodent neurotoxic reactive astrocytes. Importantly, human reactive astrocytes also lose several homeostatic functions and become potently neurotoxic to hiPSC-derived neurons, validating the findings from rodent models (Barbar et al., 2020a). This evolutionary conservation of function has also been alluded to in cross-species toxicity assays with rodent neurotoxic astrocytes able to induce apoptosis in hiPSC-derived dopaminergic neurons (Liddelow et al., 2017).

Over the past years, multiple studies from independent laboratories have demonstrated that hiPSC modeling is a powerful tool for uncovering the role of astrocytes in CNS diseases, including AD (Oksanen et al., 2017; Preman et al., 2021; de Leeuw et al., 2022), Parkinson’s disease (di Domenico et al., 2019; de Rus Jacquet et al., 2021; Ramos-Gonzalez et al., 2021; Russ et al., 2021; Trudler et al., 2021) and amyotrophic lateral sclerosis (Birger et al., 2019; Zhao et al., 2020; Szebényi et al., 2021; Ziff et al., 2021; Giacomelli et al., 2022). Developing human models is particularly critical since such diseases cannot be fully recapitulated in animal systems and thus require multiple integrated approaches (i.e., combination of human and animal studies) to understand their pathogenic mechanisms.

A recent study using hiPSC modeling has classified TIC-induced reactive astrocytes into at least two distinct subpopulations using single-cell sequencing (scRNA-seq) analyses and CRISPRi screening (Leng et al., 2021); however, deeper characterization of these subpopulations is hindered by the lack of specific markers that enable their purification. Furthermore, although several transcriptomic datasets have been generated using rodents (Clarke et al., 2018; Pan et al., 2020; Hasel et al., 2021), human models (Teh et al., 2017; Barbar et al., 2020a), and human post-mortem brain samples (Mathys et al., 2019; Zhou et al., 2020; Sadick et al., 2022), studies on the proteome of human reactive astrocytes are still limited.

To address these gaps, here we exploited our established protocol to generate hiPSC-derived astrocytes and expanded the molecular characterization of the previously described neurotoxic reactive state linked to neurodegeneration. We conducted a flow cytometry screen for surface antigens and performed proteomic profiling of TIC-induced reactive astrocytes, compared to unstimulated cells. We identified VCAM1, BST2, ICOSL, HLA-E, PD-L1, and PDPN as novel markers of TIC-induced reactive astrocytes. Notably, we found these markers were also upregulated at the transcriptional level. Whole cell proteomic analysis confirmed the upregulation of all six markers and highlighted pathways linked to engagement with peripheral immune cells. Notably, we localized several of these markers in human post-mortem brain samples from AD patients, but not from healthy individuals. Overall, our study defines reactive astrocyte-specific proteins and pathways that can be further investigated to better understand the involvement of astrocyte responses in the pathology of neurodegenerative diseases.

## Results

### Flow Cytometry Screen Identifies Novel Markers of TNF, IL-1α, and C1q-Induced Neurotoxic Reactive Astrocytes

**FIGURE 1:**
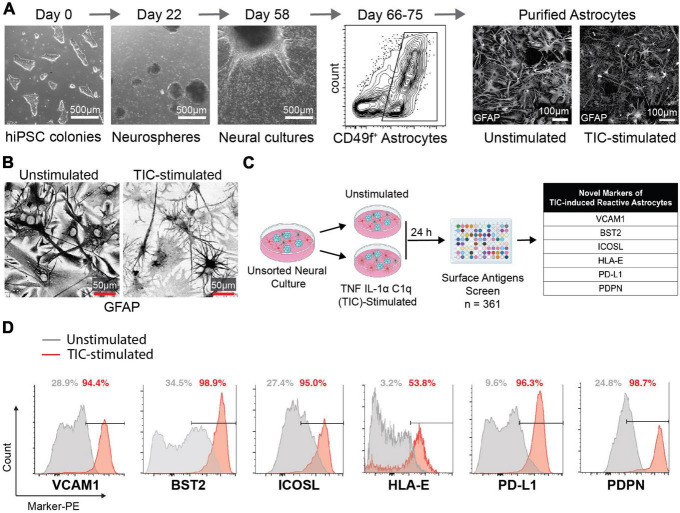
Flow cytometry screen identifies novel markers for neurotoxic TIC-induced reactive astrocytes. **(A)** Representative images of major timepoints in hiPSC differentiation protocol into astrocytes. Between days 66 and 75 astrocytes are purified using CD49f (representative flow cytometry contour plot shown). Immunofluorescence staining for GFAP in unstimulated and TIC-induced reactive astrocytes highlights the change in morphology caused by proinflammatory stimulation. **(B)** Representative images of GFAP immunofluorescence staining in unstimulated and TIC-induced reactive astrocytes at high magnification. We used inverted grayscale to emphasize the changes in morphology caused by proinflammatory stimulation. **(C)** Schematic of flow cytometry screening of 361 PE-conjugated antibodies identifies six novel markers of TIC-induced reactive astrocytes, listed in the table. **(D)** Flow cytometry detection of each marker from the screen is shown as histogram plot comparing TIC-induced reactive (red) to unstimulated (gray) astrocytes.

We first performed a flow cytometry screen of surface molecules to identify antigens that may enable isolation of reactive astrocytes for downstream analyses. We differentiated hiPSCs to neural progenitor cells that self-assembled into neurospheres and then plated the spheres onto poly-ornithine/laminin-coated dishes to allow migration and differentiation into astrocytes (Barbar et al., 2020a; Figure 1A; Supplementary Figure 1). After 66–75 days, the cells that migrated out of the neurospheres formed a monolayer composed of about 50% astrocytes, 40% oligodendrocyte lineage cells, and 10% neurons. Next, we stimulated these cultures with TIC for 24 h, leaving some wells as unstimulated controls. Through immunofluorescent staining for glial fibrillary acidic protein (GFAP), we observed a drastic change in morphology of TIC-induced reactive astrocytes when compared to the unstimulated controls (Figure 1B) as we have previously reported (Barbar et al., 2020a). Of note, elevated GFAP staining was found in both unstimulated and TIC-induced reactive astrocytes, supporting previous findings attributing increased GFAP levels to *in vitro* culture conditions (Molofsky et al., 2012). We then screened 361 surface molecules, gating on the CD49f^+^ population (Supplementary Figure 2) and selected six surface molecules that were enriched in the TIC-induced reactive astrocytes for further investigation: VCAM1, BST2, ICOSL, HLA-E, PD-L1, and PDPN (Figure 1C). Using histogram plots we showed the enrichment of each of these markers within the TIC-induced reactive astrocyte population compared to the unstimulated cells (Figure 1D).

**FIGURE 2:**
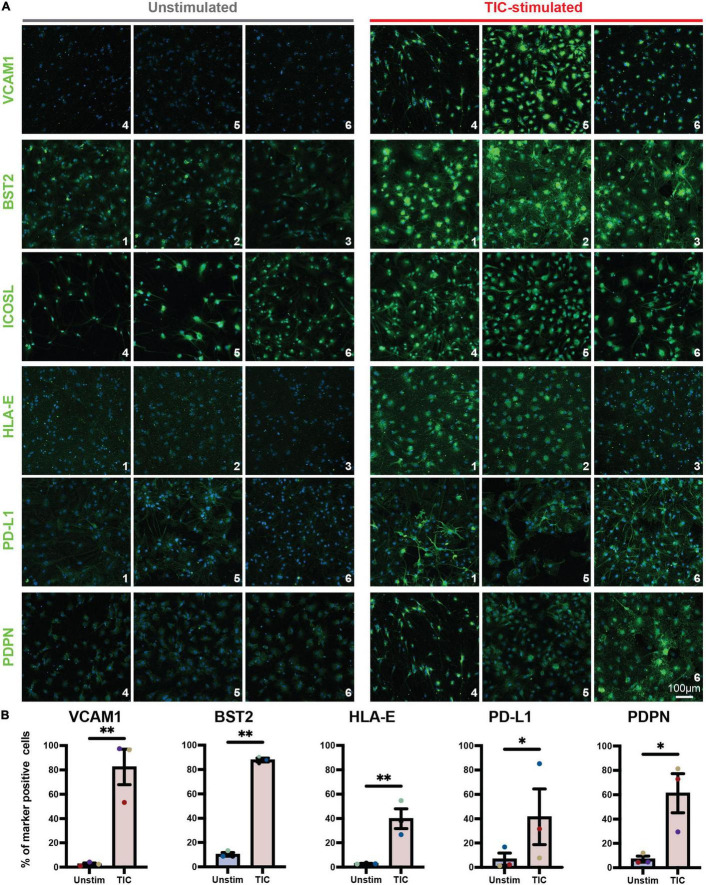
Immunofluorescence analysis of novel TIC-reactive markers in CD49f^+^-sorted astrocytes. **(A)** Independent immunofluorescence analysis on CD49f-purified astrocytes using antibodies conjugated with fluorescent secondaries. All markers (with the exception of ICOSL) are enriched in TIC-stimulated astrocytes, validating the flow cytometry findings. Each marker is shown in AF488 (green) and nuclei are labeled with Hoechst 33342 (blue). Each marker was tested on astrocytes derived from three hiPSC lines (*n* = 3); line code numbers are shown in the bottom right corner of each image. **(B)** Quantification of marker-positive cells shown as percentage of total cells (all nuclei). Error bars represent standard error of means. *p*-value was calculated using ratio paired *t*-test. *, *p*-value < 0.05; **, *p*-value < 0.01.

**FIGURE 3:**
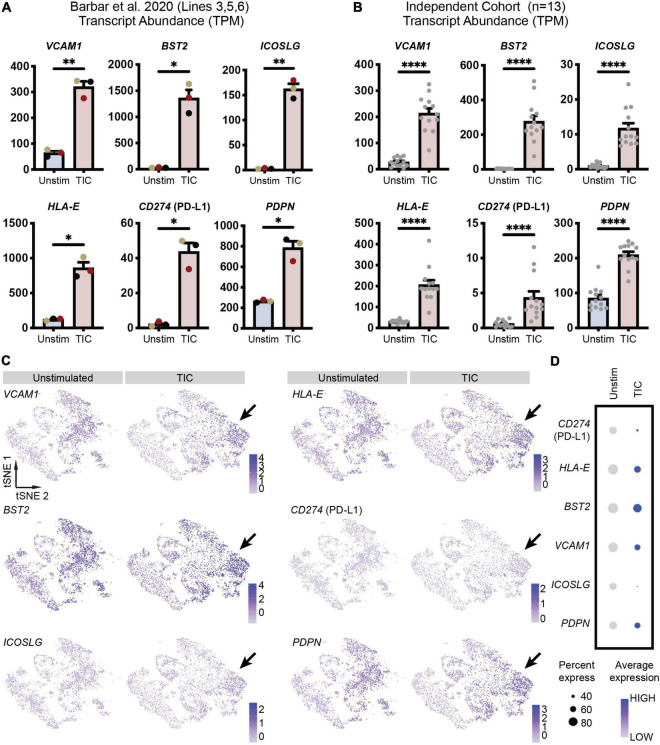
Novel reactive astrocyte markers are upregulated at the transcriptional level. **(A)** Transcript abundances in transcripts per million (TPM) for each of the six genes are plotted to visualize marker expression levels for each individual line (*n* = 3, line 3 = black, line 5 = yellow, line 6 = red from Barbar et al., 2020a). Individual dots are means of three technical replicates for each line; error bars represent standard error of means. *p*-value was calculated using a two-tailed Welch’s *t*-test. *, *p*-value < 0.05; **, *p*-value < 0.01. **(B)** Transcript abundances (in TPM) for each of the six genes are plotted to visualize marker expression in hiPSC-derived astrocytes from an independent cohort of 13 lines (individual replicates shown as dots). Error bars represent standard error of means. *P*-value was calculated using a Wald test through DESeq2 and adjusted for multiple comparisons using the Benjamini and Hochberg method. ****, *p*-value < 0.0001. **(C)** Feature plots of CD49f^+^ hiPSC-derived astrocyte scRNA-seq (Barbar et al., 2020a) demonstrates increased expression of each marker’s associated gene in TIC-induced reactive astrocytes compared to unstimulated astrocytes. Arrows highlight clusters most associated with TIC-stimulation and having the greatest upregulation of putative reactive astrocyte markers. **(D)** Dot plot showing genes enriched in TIC-induced reactive astrocytes and unstimulated astrocytes. Each row is an individual marker gene.

To confirm screening results, we next performed an independent immunofluorescence assay on a second batch of differentiations; in this experiment, we used CD49f-sorted astrocytes rather than mixed cultures to eliminate any non-astrocyte responses to the inducing stimuli. As shown in Figure 2A, all six proteins were detected in the TIC-stimulated condition. Quantification of immunofluorescence staining for VCAM1, BST2, HLA-E, PD-L1, and PDPN confirmed a significantly greater number of positive cells upon stimulation across astrocytes from three independent hiPSC lines from healthy donors (Figure 2B). Next, leveraging our available RNA-sequencing datasets (Barbar et al., 2020a), we probed for each of the six genes encoding for the markers to assess whether they were upregulated at the mRNA level. All genes were upregulated within the three hiPSC-line previously used (Figure 3A); notably, these findings were confirmed when we extended the analysis to an independent cohort of an additional 13 hiPSC lines that were differentiated to astrocytes in a parallel study (Figure 3B). We also probed for these six genes within our available scRNA-sequencing datasets of TIC-induced reactive versus unstimulated astrocyte cultures (Barbar et al., 2020a; Figure 3C). Focusing on the astrocyte clusters, we found that expression of each gene is distinctly higher in the TIC-induced reactive condition, which confirms that VCAM1, BST2, ICOSL, HLA-E, PD-L1, and PDPN are upregulated upon stimulation, with BST2 being the most upregulated and ICOSL being the least (Figure 3D).

We therefore conclude that VCAM1, BST2, ICOSL, HLA-E, PD-L1, and PDPN are highly expressed by hiPSC-derived astrocytes in response to TIC stimulation and we propose them as putative, novel markers of TIC-induced reactive astrocytes.

### Proteomic Profiling of TNF, IL-1α, and C1q-Induced Reactive Astrocytes Underscores Their Putative Involvement in Immune Responses

**FIGURE 4:**
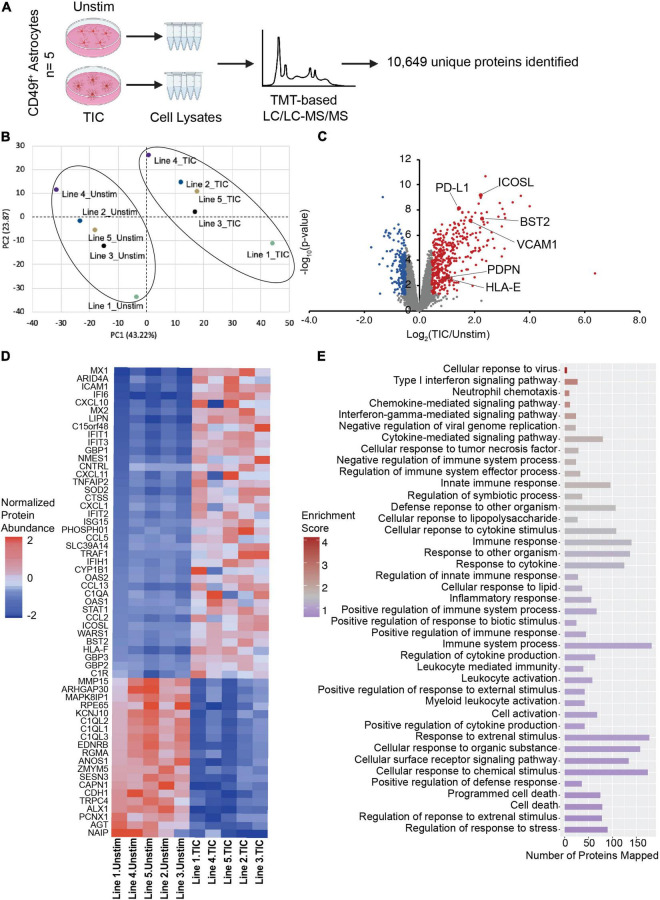
Proteomic analysis implicates TIC-stimulated reactive astrocytes interaction with peripheral immune cells and neurotoxic biological processes. **(A)** Schematic of proteomic analysis workflow performed with TIC-induced reactive versus unstimulated astrocytes. **(B)** Principal component analysis using top 10% most variable proteins displays clustering of unstimulated and TIC-stimulated astrocytes. **(C)** Volcano plot of differentially expressed proteins. Proteomic analysis further validated the six markers identified through flow cytometry screen, which are highlighted. For significance threshold, *P*-value < 0.05 and *z*-scores > 2 were used. **(D)** Heatmap of top 10% most variable proteins in TIC-induced reactive versus unstimulated astrocytes. **(E)** Pathway enrichment analysis using 601 differentially expressed proteins highlights biological processes involved in immune response and inflammatory cascades.

We have previously shown that human and rodent cells have a highly conserved transcriptomic signature upon TIC stimulation (Barbar et al., 2020a). Although transcriptomic analyses are powerful tools, gene expression does not always correlate with protein abundance (Leitner et al., 2021; Johnson et al., 2022). Thus, we extended the characterization of our human model to include proteomic profiling of TIC-induced reactive *versus* unstimulated astrocytes derived from five independent hiPSC lines from healthy donors (Figure 4A). We confirmed high protein quality and no loading bias (Supplementary Figure 3A) and performed Tandem Mass Tag-based (TMT) liquid chromatography/liquid chromatography-mass spectrometry/mass spectrometry (LC/LC-MS/MS; Bai et al., 2017), which identified 10,649 unique proteins. Of these, 601 proteins were differentially expressed (DE; 400 upregulated and 201 downregulated; Supplementary Table 1) using the thresholds of *p*-value < 0.05 and absolute *z*-score > 2. Furthermore, principal component analysis (Figure 4B) of the top 10% DE proteins showed a clear separation of the TIC-treated and unstimulated astrocytes, indicating a dramatic change to the astrocyte proteome upon TIC stimulation. Hierarchical clustering of the DE proteins and samples emphasized the robust and highly consistent response to proinflammatory stimuli across all lines, with some line-to-line variability (Supplementary Figure 3B). We also examined the six markers identified through flow cytometry and further confirmed their increased abundance in TIC-induced reactive astrocytes (Figure 4C), with BST2 and ICOSL showing the greatest fold change. We then probed a recent proteomic dataset of TIC-induced reactive rat astrocytes (Guttenplan et al., 2021) and found that VCAM1 and RT-BM1 [RT1.A1(F) domain; ortholog of HLA-E] were more abundant in rat TIC-induced reactive astrocytes compared to unstimulated cells, indicating these two as potentially conserved markers (Supplementary Figure 4). The other human markers were not detected in the rat dataset using this methodology.

Of the top 10% most DE proteins detected in our human system (Figure 4D), many were known chemokines (e.g., CXCL10, CXCL11) and interferon inducible proteins (e.g., IFI6, IFIT1, IFIT3). Pathway enrichment analysis based on the DE proteins revealed a total of 67 significantly enriched GO-terms (Supplementary Table 2), mostly related to immune responses, cytokine-associated signaling, and recruitment of peripheral immune cells to the CNS (Figure 4E).

Taken together, these results suggest that the proteome of hiPSC astrocytes undergoes rapid and robust changes upon TIC stimulation. The proteomic profiling of reactive astrocytes suggests a putative participation in immune responses and in the processes of neuroinflammation associated with CNS diseases, while independently validating the novel surface markers.

### Reactive Astrocyte Cell Surface Markers Are Enriched in Human Alzheimer’s Disease Post-mortem Brain

**FIGURE 5:**
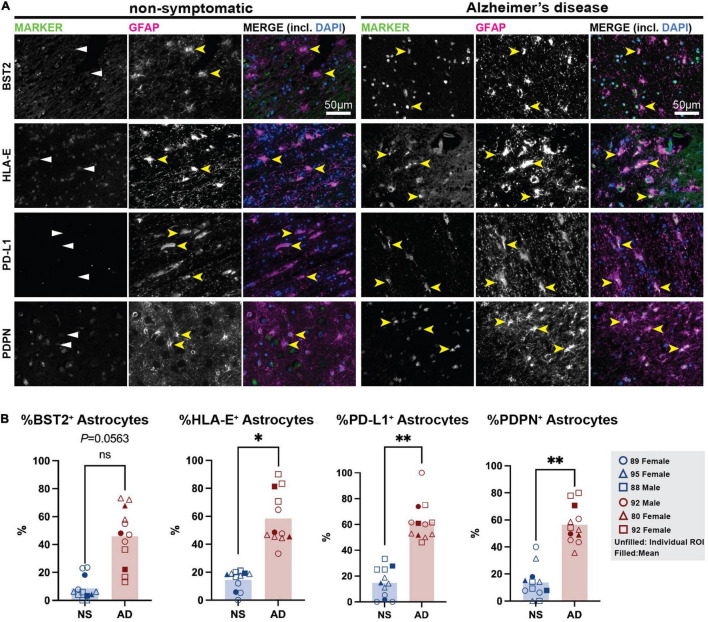
Immunostaining for putative TIC-induced reactive astrocyte surface markers in human post-mortem brain. **(A)** Non-symptomatic (left) and Alzheimer’s disease (right) cortical sections were stained for BST2, HLA-E, PD-L1, and PDPN (all in green), and co-stained for the canonical astrocyte marker GFAP (magenta), and nuclear stain DAPI (blue). Yellow arrowheads mark astrocytes positive for GFAP (in NS brains) and/or specific cell surface reactive astrocyte marker (in Alzheimer’s brain samples). White arrowheads mark absence of reactivity markers in non-symptomatic brain. Scale bar = 50 μm. **(B)** Quantification of BST2, HLA-E, PD-L1, and PDPN in human tissues. Plots show the individual points from each ROI (unfilled shapes), with means shown as filled shapes. Data are shown as percentage of double positive (Marker^+^/GFAP^+^) cells over GFAP^+^ astrocytes. *, *p*-value < 0.05; **, *p*-value < 0.01.

Next, we sought to confirm the presence of these markers in the astrocytes in human post-mortem brain samples. We selected a cohort of AD patient and age-matched non-symptomatic (NS) controls (Sadick et al., 2022) and stained formalin-fixed paraffin embedded frontal cortex samples for our putative reactive astrocyte markers. Broadly, we report no or minimal immunofluorescence for any marker in the NS brain. In the AD brain, we observed that PD-L1^+^ cells were almost exclusively GFAP^+^ in the white matter, suggesting this marker is largely restricted to astrocytes in this region (Figure 5 and Supplementary Figure 5). Another marker, PDPN, was also almost exclusively found in GFAP^+^ astrocytes in the AD brain, while some GFAP^–^ cells were positive for PDPN in the NS brain. Similarly, BST2 protein was abundant in the AD tissue compared to NS tissue; however, its presence was not limited to GFAP^+^ astrocytes. This could indicate the presence of BST2^+^ non-astrocytic cells in the human brain, but it must be noted that GFAP astrocytes exhibit regional heterogeneity and some of the BST2 signal observed in these regions could possibly be in these GFAP^–^ astrocytes. Finally, astrocytes in the AD tissue showed increased HLA-E toward the sub-pial regions of the layer 1 of the prefrontal cortex. However, HLA-E was also enriched near the blood vessels and outside GFAP^+^ astrocytes in these regions. Together, these preliminary investigations suggest that cell surface markers identified in hiPSC-derived reactive astrocyte cultures are able to detect populations of astrocytes in the human post-mortem brain, with enrichment in the AD brain.

## Discussion

Human iPSC modeling has quickly become a critical complementary tool to animal models for understanding complex CNS disorders (Preman et al., 2021; Rivetti di Val Cervo et al., 2021). Efficient protocols to generate the main CNS cell types (neurons, microglia, astrocytes, oligodendrocytes, etc.) are now available to investigate cell-intrinsic mechanisms linked to physiological functions and dysfunctions in disease pathology (Hu et al., 2009; Chambers et al., 2012; Maroof et al., 2013; Douvaras and Fossati, 2015; Abud et al., 2017; Douvaras et al., 2017; Tcw et al., 2017; Perriot et al., 2018; Barbar et al., 2020b; Guttikonda et al., 2021). Findings from human *in vitro* models provide the foundation for subsequent investigations in more complex systems, such as human brain organoids and *in vivo* animal models. The integration of all these systems, coupled with the advancing technologies for single-cell resolution, will greatly expand our understanding of reactive astrocyte functions and biology and can propel the development of precise and effective therapies for neurodegenerative diseases where inflammation is a major contributor. Here, we used a model of purified hiPSC-derived astrocytes, that—eliminating all interactions with other cell types—allowed us to focus on the intrinsic changes that these cells undergo upon activation to a neurotoxic reactive state, driven by inflammation (TIC). The serum-free conditions used in all our cultures were critical to generate astrocytes similar in morphology and function to their *in vivo* counterparts and to mimic *in vitro* the transition into a reactive state that we and others have reported in rodent astrocytes both *in vitro* and *in vivo*. The reactive response to TIC stimulation observed *in vitro* was remarkably consistent across astrocytes derived from six hiPSC lines that were used in this study and 13 additional lines from an independent cohort, demonstrating the robustness of our system. Through a flow cytometry screen, we identified VCAM1, BST2, ICOSL, HLA-E, PD-L1, and PDPN, which together define a signature for hiPSC-derived reactive astrocytes. Interestingly, we found a greater number of BST2, HLA-E, PD-L1, and PDPN positive GFAP^+^ astrocytes in AD post-mortem brains compared to brains from non-symptomatic individuals, warranting further investigations on the roles that these markers play in neurodegenerative disease. While flow cytometry, transcriptomic, and proteomic analyses clearly identified significantly higher levels of these markers upon TIC stimulation, quantification of positive cells through staining both *in vitro* and *in vivo* was in some cases hindered by the lack of validated and commercially available antibodies. In particular, ICOSL antibody stained all cells in both unstimulated and stimulated conditions, even after testing different antibodies and fixation protocols. This result is in contrast with proteomic, transcriptomic, and flow cytometry analyses; thus, we concluded that immunofluorescent staining for ICOSL in astrocytes and brain samples is not reliable and additional troubleshooting will be required for immunofluorescence and immunohistochemical analyses. Nonetheless, we believe these markers may be useful for dissecting reactive astrocyte states and allow for the resolution of the spatiotemporal characteristics of reactive astrocytes during CNS disease onset and progression. The six molecules have been previously studied in different contexts and we have outlined the literature summarizing their known functions in Table 1. Their specific function in reactive astrocytes remains elusive; however, future investigation into the functions of each protein and the pathways in which they are involved may reveal novel roles of reactive astrocytes in the context of infection, injury, and disease.

**TABLE 1:** Publications describing known functions of novel reactive astrocyte markers.

Protein name (Alternate names)	Known functions	Role in astrocytes
VCAM1–Vascular Cell Adhesion Molecule 1 (CD106)	Gimenez et al., 2004; Ou et al., 2008; Haarmann et al., 2015; Kong et al., 2018; Pinho et al., 2022	Cheng et al., 2019; Leng et al., 2021
BST2–Bone Marrow Stromal Cell Antigen 2 (Tetherin; CD317)	Jouvenet et al., 2009; Perez-Caballero et al., 2009; Cocka and Bates, 2012; Dafa-Berger et al., 2012	Ponath et al., 2018
ICOSL–Inducible T-Cell Costimulator Ligand (ICOSLG; CD275)	Vocanson et al., 2010; Choi et al., 2011; Wikenheiser and Stumhofer, 2016; Iwata et al., 2019; Saravia et al., 2020	Li et al., 2018; Recasens et al., 2018
HLA-E–Major Histocompatibility Complex I, E (MHC Class I Antigen E)	Coupel et al., 2007; Rölle et al., 2018	Morandi et al., 2013; Lau et al., 2020; Wu et al., 2020
PD-L1–Programmed Cell Death 1 Ligand 1 (CD274; B7H1)	Freeman et al., 2000; Cha et al., 2019; Doroshow et al., 2021	Pittet et al., 2011; Kummer et al., 2021
PDPN–Podoplanin	Astarita et al., 2012; Martín-Villar et al., 2015	Kolar et al., 2015; Li et al., 2018

Vascular cell adhesion protein 1 (VCAM1) was recently identified by an independent study as an *in vitro* TIC-induced reactive astrocyte marker (Leng et al., 2021). Increased VCAM1 levels was also reported in rat TIC-induced reactive astrocytes *in vitro* (Guttenplan et al., 2021), likely making it an evolutionarily conserved marker that can be used to isolate reactive astrocytes in both rodent and human samples. An important role of VCAM1 is the recruitment of circulating leukocytes by activated endothelial cells to sites of tissue inflammation (Alon et al., 1995; Berlin et al., 1995; von Andrian and Mackay, 2000). Although it remains unclear whether VCAM1 is elevated in human astrocytes *in vivo*, there is evidence supporting that it is expressed within demyelinated lesions in mouse models of experimental autoimmune encephalitis and MS (Gimenez et al., 2004). Soluble VCAM1 has also been implicated in the disruption of the blood-brain barrier (Haarmann et al., 2015). It is unclear how astrocytic VCAM1 may play a role in modulating inflammation, and it remains an interesting target to understand the mechanisms by which peripheral immune cells infiltrate the brain in CNS diseases—especially in AD (Cao and Zheng, 2018). Similarly, ICOSL (Inducible T Cell Costimulator Ligand) is likely implicated with CNS-infiltration of peripheral immune cells. It is the ligand for costimulatory receptor ICOS on T cells (Hutloff et al., 1999; Tafuri et al., 2001; Nurieva et al., 2009; Choi et al., 2011). ICOSL-ICOS signaling has also been implicated in regulatory T cell functions (Vocanson et al., 2010; Kornete et al., 2012; Iwata et al., 2019; Li and Xiong, 2020; Saravia et al., 2020). Furthermore, in a previous collaboration, we detected upregulation of *ICOSLG* at the transcriptional level in both post-mortem brain samples and hiPSC-derived astrocytes from patients with Alexander Disease, a neurodegenerative disease caused by mutations in *GFAP* (Li et al., 2018).

Another of our reactive astrocyte surface markers, podoplanin (PDPN), a mucin-type transmembrane protein, closely associates with tumors of the brain and peripheral organs (for review see Astarita et al., 2012). This cell-adhesion molecule has been found to colocalize with subpopulations of GFAP^+^ reactive astrocytes in mouse models of brain injury and glioblastoma. It is also upregulated at the mRNA level in both post-mortem brain samples and hiPSC-derived astrocytes from Alexander Disease patients (Li et al., 2018). In addition, PDPN is a known regulator of TH17 cells, which play an important role in the pathophysiology of autoimmune diseases such as MS (Nylander et al., 2017). Another molecule that is linked to several immune functions is bone marrow stromal cell antigen 2 (BST2). Although we did not find studies on this protein’s expression specifically in astrocytes, the longer isoform of BST2 induces NF-κB signaling (Cocka and Bates, 2012). NF-κB is a central mediator of the priming signal and regulator of innate immunity, and is a well-known pathway in astrocyte reactivity responses and has been implicated in lymphocyte recruitment to lesions in CNS diseases (Ponath et al., 2018).

HLA-E and PD-L1 (encoded by *CD274*) have both been extensively studied in regulation of immune cell activation (Freeman et al., 2000; Coupel et al., 2007). In the CNS, *HLA-E* expression was found to be upregulated in post-mortem brain samples from people with AD (Lau et al., 2020) and primary diffuse gliomas (Wu et al., 2020). HLA-E protein abundance is higher in relapsing remitting MS patients compared to patients with other non-MS inflammatory disorders (Morandi et al., 2013). MS patients also present with increased *CD274* expression–particularly in astrocytes and microglia (Pittet et al., 2011). A recent study implicated soluble PD-L1 cleaved from astrocyte membrane binds to microglial PD-1 receptors, driving phagocytosis of amyloid plaques and inhibiting plaque growth in models of AD and in human AD patients–suggesting a key protective role of reactive astrocytes in disease (Kummer et al., 2021). Future investigations should explore whether astrocytic PD-L1 may have a similar protective role in MS and other neurological diseases.

Overall, the signature defined by these six reactive astrocyte surface markers highlights an important putative feedback from reactive astrocytes to immune cells in inflammatory conditions. As part of their critical physiological functions, astrocytes have been described as antigen-presenting cells with the capacity to present peptide antigens in cell-surface MHC molecules and expressing immunomodulatory molecules (Massa et al., 1993; Stüve et al., 2002; Priego and Valiente, 2019; Rostami et al., 2020). Upon stimulation, reactive astrocytes have been associated with recruitment of peripheral immune cells to the CNS and secretion of factors that promote microglial activation in a positive feedback loop (Guttikonda et al., 2021; McAlpine et al., 2021); these functions are further supported by our proteomic results, as we report a significant upregulation of chemokines such as CXCL10, CXCL11, and complement component 3 (C3). Indeed, CXCL10 is implicated in other sub-states of reactive astrocytes, particularly those driven by interferon signaling in the mouse (Hasel et al., 2021) and visualized in human AD (Xia et al., 2000) and MS (Sørensen et al., 1999) post-mortem samples. Moreover, pathway enrichment analysis showed that the vast majority of the upregulated biological processes of TIC-induced reactive astrocytes were implicated with immune responses and recruitment of peripheral immune cells (a key function of CXCL10).

In conclusion, we used our hiPSC-based system to define the proteomic signature of TIC-induced reactive astrocytes *in vitro* and compared this to quiescent, non-reactive astrocytes. Such discoveries in *in vitro* systems are integral to drive future studies that investigate the functional relevance of our findings *in vivo* through spatial and temporal characterizations in various CNS disease contexts, both in animal models and in human specimens. In addition, functional testing of reactive astrocyte sub-states is currently not possible *in vivo*, so serum-free, pure cultures of reactive astrocyte sub-states *in vitro* are essential to uncover novel functions of astrocytes in health and disease. These functions can be further elucidated by developing more sophisticated co-cultures or organoid cultures that include other neural cells (e.g., neurons, oligodendrocytes) and/or immune cells (microglia, leukocytes). Future studies will also enable investigation of individual and disease-specific variations in reactive astrocyte responses through use of automated platforms for population-scale hiPSC modeling (Lagomarsino et al., 2021). The integration of this high-throughput technology with *in vivo* studies in animal systems will be crucial to dissect the molecular mechanisms of glia-mediated toxicity across different CNS diseases and to ultimately accelerate discoveries toward treatments.

## Materials and Methods

### Human Induced Pluripotent Stem Cell Lines

In this study, we used six hiPSC lines from healthy donors, that were previously generated using the NYSCF Global Stem Cell Array^®^, a fully automated platform for fibroblast and blood reprogramming (Paull et al., 2015). All lines passed stringent quality controls, including karyotyping, mycoplasma testing, pluripotency efficiency, and identity match. A certificate of analysis from one of the lines is shown in Supplementary Figure 6. Five of these lines were used in our previous studies on hiPSC-derived astrocytes (Barbar et al., 2020a,b). Table 2 contains the demographic information for the donors of the skin biopsies that were reprogrammed to hiPSCs.

**TABLE 2:** Identification code assignment and demographics of hiPSC-line donors.

Line code	Original line ID	Age at biopsy	Sex	Flow cytometry screen	Bulk RNA-sequencing	Single-cell RNA-sequencing	Whole proteome analysis	Immunofluorescence
1	051064-01-MR-007	53	male				X	BST2; HLA-E; PD-L1
2	051104-01-MR-040	56	female				X	BST2; HLA-E; PD-L1
3	051121-01-MR-017	52	female		X	X	X	BST2; HLA-E; PD-L1
4	050659-01-MR-013	64	female				X	VCAM1; ICOSL; PDPN
5	050743-01-MR-023	50	male		X	X	X	VCAM1; ICOSL; PDPN
6	051106-01-MR-045	57	female	X	X			VCAM1; ICOSL; PDPN

### Human Induced Pluripotent Stem Cell Differentiation Into Astrocytes and Stimulation to a Reactive Astrocyte State

All cultures were maintained in a 37°C incubator with 5% CO_2_. For astrocyte differentiation, hiPSCs were plated at 1.5 × 10^5 cells per well in a 6-well geltrex-coated plate and fed with mTeSR1 maintenance medium with 10 μM Y27632 for the first 24 h after seeding (Marotta et al., 2021). Differentiation and FACS-purification of CD49f^+^ astrocytes were performed using a serum-free protocol that we have previously established; differentiation media and patterning reagents are fully detailed in our previous publications and summarized in a schematic of the protocol in Supplementary Figure 1 (Barbar et al., 2020a,b). After enzymatic dissociation of neural cultures for fluorescence-activated cell sorting on days 66–75, neurospheres were replated and isolation of CD49f^+^ astrocytes was repeated on Day 95–105 to isolate “second round spheres,” as previously detailed (Barbar et al., 2020a). To model a neurotoxic reactive astrocyte state, mixed neural cultures and sorted CD49f^+^ astrocytes around day 65 were stimulated for 24 h with human recombinant proteins TNF (30 ng/mL, R&D Systems; 210-TA-020), IL1α (3 ng/mL, Sigma; 3901), and C1q (400 ng/mL, MyBioSource; MBS143105), which we defined as “TIC.”

### Immunofluorescence Staining of Human Induced Pluripotent Stem Cell-Derived Astrocytes

Astrocytes from three lines were fixed using 4% paraformaldehyde for 10 min then washed 3× with DPBS. Cells were then incubated for one hour in blocking solution (DPBS + 5% donkey serum + 0.1% Triton-X-100) followed by overnight incubation with primary antibody at 4°C. After 24 h, cells were washed 3× with DPBS followed by incubation for 1 h with secondary antibody (Alexa Fluor) at 1:1000 dilution and washed 3× with DPBS. Cells were then incubated with HOECHST 33342 (1:1000 dilution) for 10 min and washed 3× with DPBS. Images were acquired on Zeiss scanning confocal microscope (LSM800). All antibodies used are listed in the Table 3.

**TABLE 3:** Antibodies for immunohistochemistry.

Antibody	Sample	Dilution	Vendor	Cat. no.
PD-L1	hiPSC-astrocytes and post-mortem samples	1:50	R&D Systems	MAB1561
HLA-E	hiPSC-astrocytes and post-mortem samples	1:50	Abcam	Ab2216
BST2	hiPSC-astrocytes and post-mortem samples	1:100	Abcam	Ab88523
VCAM1	hiPSC-astrocytes and post-mortem samples	1:250	Abcam	Ab134047
ICOSL	hiPSC-astrocytes and post-mortem samples	1:100	Abcam	Ab233151
PDPN	hiPSC-astrocytes and post-mortem samples	1:250	BioLegend	337001
GFAP	hiPSC-astrocytes	1:1000	EMD Millipore	MAB360
Rabbit anti-GFAP	post-mortem samples	1:500	Agilent Dako	Z0334
Donkey Alexa Fluor 488 and 647 secondary	hiPSC-astrocytes	1:1000	ThermoFisher	A-21202 A-31571
Goat Anti-Mouse Alexa Fluor 488	post-mortem samples	1:300	Abcam	ab150113
Goat Anti-Rabbit Alexa Fluor 488	post-mortem samples	1:300	Invitrogen	A11034
Goat Anti-Rabbit Alexa Fluor 594	post-mortem samples	1:300	Invitrogen	A11012
Goat Anti-Rat Alexa Fluor 488	post-mortem samples	1:300	Invitrogen	A11006

### Quantification of Immunofluorescence Staining

Astrocytes fixed and stained for each marker were imaged on the Opera Phenix High-Content Screening System (PerkinElmer, Waltham, MA, United States). Images were acquired at 10× magnification and stitched together to capture nearly the entirety of each well (*n* = 9 fields per well) while maintaining consistent imaging settings for each marker across lines. Harmony analysis software was used to identify nuclei of live cells using size and HOECSHT 33342 intensity criteria to exclude apoptotic nuclei and debris. The nuclear region was then expanded by 50% to create a region of interest (ROI) and the Alexa Fluor intensity within this region in a separate channel was quantified within each ROI. Thresholds were set consistently for each marker across all lines, with ROIs exceeding the threshold identified as astrocytes positive for the marker. Results were reported as percentage of positive astrocytes out of all live cells (HOECHST nuclei) identified.

### Flow Cytometry Screen for Cell Surface Antigens

We performed a cell surface antigen screen on cells differentiated from hiPSC line 6 (Line ID 051106). Cultures at day 64 were enzymatically digested with accutase (ThermoFisher, Waltham, MA, United States; A1110501) for 20 min at 37°C and second round spheres were re-plated to continue differentiation as described (Barbar et al., 2020a). At day 95, cells were stimulated with TIC cocktail for 24 h. Unstimulated wells were also prepared as control. Cells were then dissociated using accutase for 30 min at 37°C, diluted in DMEM, and filtered using a 70 μm filter (STEMCELL Technologies; 27260) to eliminate spheres and cells aggregates. The cell suspension was centrifuged at 300 g for 5 min and resuspended in FACS buffer (DPBS + 0.5% BSA + 1% penicillin/streptomyocin + 2mM EDTA + 20 mM glucose). For barcoding, TIC-induced astrocytes were incubated for 15 min at room temperature (RT) with live/dead fixable near-IR (ThermoFisher; L34976), washed 2× with FACS Buffer, and centrifuged at 300 *g* (RT) for 5 min. Unlabeled (unstimulated) and barcoded (TIC-stimulated) cells were then combined and stained with CD49f-Pacific Blue (1:25 dilution, BioLegend; 313619) and incubated for 20 min at 4°C. Cells were washed 1× with FACS buffer and centrifuged at RT and 300 *g* for 5 min. Cells were distributed evenly into four 96-well plates of the LEGENDScreen™ Human PE kit (Biolegend; 700007), containing 361 PE-conjugated antibodies to cell surface markers. Plates were incubated for 20 min at 4°C, washed 1× with FACS buffer, and resuspended in each well. Samples were acquired on a 4-laser Attune NxT Flow Cytometer (405, 488, 561, 633, Thermo Scientific). Subsequent data analysis was performed in FlowJo v.10.

### Gene Expression of Novel Surface Markers

mRNA levels for the six markers of TIC-induced reactive astrocytes were retrieved from our previous bulk transcriptome analysis performed on CD49f^+^ TIC-induced reactive versus unstimulated astrocytes, differentiated from lines 3, 5, and 6 (Barbar et al., 2020a).

### Gene Expression of Novel Surface Markers in an Independent Cohort of Human Induced Pluripotent Stem Cell Lines

mRNA levels for the six markers of TIC-induced reactive astrocytes were also retrieved from an independent cohort of 13 hiPSC lines from MS patients and age/sex matched controls. These lines were reprogrammed using the NYSCF Global Stem Cell Array^®^, from peripheral blood mononuclear cells and they all passed the quality controls described above. Human iPSC lines were differentiated to CD49f-astrocytes and stimulated to a TIC-induced reactive state as described above. Details can be found in a manuscript published back-to-back to the special issue “Glia-mediated neurotoxicity: uncovering the molecular mechanisms” (Smith et al., 2020). Cells were washed and lysed in RLT Plus buffer (Qiagen RNeasy Plus Micro Kit #74034) and lysates were frozen at −80°C. RNA was isolated following manufacturer’s recommended protocol. RNA quality was assessed on a Fragment Analyzer to proceed to library preparation. Total RNA libraries were generated using Illumina Stranded Total RNA Prep with Ribo-Zero Plus kit. Libraries were sequenced (100 bp paired end) on an Illumina NovaSeq. Sequencing data quality was checked with FastQC (Andrews, 2010) followed by MultiQC (Ewels et al., 2016). Transcript quantification was done with Salmon (v1.4.0) (Patro et al., 2017) against RefSeq GRCh38.p12 reference genome (Schneider et al., 2017) in a decoy aware fashion using the entire genome as the decoy sequence (Srivastava et al., 2020). These transcript quantifications were imported into R (4.1.2) and aggregated to gene level using tximeta (v1.12.3) (Love et al., 2020). Using the gene level quantifications, library size normalization and differential expression testing was done using DESeq2 (v1.34.0) (Love et al., 2014) where donor was a blocking factor and stimulation (unstimulated versus TIC-stimulated) was the comparison variable. *P* values were calculated by DESeq2 then adjusted for multiple comparisons using the Benjamini and Hochberg method.

### Single-Cell RNA Expression of Novel Surface Markers

We assessed mRNA expression of the six surface markers from our previous analysis on astrocytes derived from lines 3 and 5 (Barbar et al., 2020a). We loaded the RStudio objects associated with Barbar et al. (2020a); see methods of that paper for detailed bioinformatic information. We then assessed mRNA expression of the six surface markers *via* the FeaturePlot and DotPlot commands in the Seurat software package (v4.0.1), separating cells by A0/A1 labels generated as part of Barbar et al. (2020a) and feature plots were generated.

### Cell Preparation and Protein Isolation for Whole Proteome Analysis

Five hiPSC lines were differentiated for proteomics. Human iPSCs were differentiated into astrocytes as described above. FACS-purification of CD49f^+^ astrocytes was performed on D75 from all lines. Table 4 details yield of CD49f^+^ astrocytes per line. CD49f^+^ astrocytes were plated into two wells of a poly-Ornithine/g-coated 24-well Nunclon-Delta plate in PDGF medium. Cells were fed the day after sorting (Day 1 post-sort) with 2/3 media change of Glial medium. On Day 3 post-sort, 2/3 medium change with Glial medium was completed. On Day 5 post-sort, full media change with Glial medium for unstimulated condition and full medium change with Glial medium plus TIC cocktail for TIC-stimulated condition. After 24 h cells were washed once with ice-cold DPBS then lysed in the well using lysis buffer containing 50 mM HEPES (Thermo Scientific; 15640080, pH = 8.5), 8M Urea (Sigma, U5378), 0.5% sodium deoxycholate (Sigma; 30970), 1 cOmplete mini EDTA-free Protease Inhibitor Cocktail tablet (Sigma; 11836170001) per 10 mL and 1 PhosSTOP tablet (Sigma; 4906845001) per 10 mL. Lysates were collected in Protein LoBind tubes (Eppendorf; 022-43-108-1) and frozen at −80°C.

**TABLE 4:** Human induced pluripotent stem cell -derived astrocytes collected for whole cell proteomic analysis.

Line code	CD49f^+^ yield
1	1,710,801
2	1,448,190
3	1,621,875
4	681,150
5	1,684,754

Protein concentration of the cell lysate was estimated by Coomassie-stained short SDS gel with BSA as a standard. Each sample was digested and TMT labeled using our previously optimized protocol (Xu et al., 2009; Wang et al., 2020) with Lys-C (Wako, 1:100 w/w) at 21°C for 3 h, then diluted with 50 mM HEPES (pH 8.5) to decrease urea concentration to 2 M, and further digested by trypsin (Promega, 1:50 w/w) at 21°C overnight. The digested peptides were reduced by dithiothreitol (DTT, 1 mM) for 30 min, alkylated by iodoacetamide (IAA, 10 mM) at dark for 30 min, and quenched with DTT (30 mM) for 30 min. The digested peptides were desalted with a C18 Micro SpinColumn (Harvard apparatus) and dried by speedvac. The dried samples were re-dissolved in 50 mM HEPES, pH 8.5 (∼2 μg/μL), reacted with 150 μg of TMTpro reagents for 30 min, then quenched with 0.5% hydroxylamine for 15 min. The labeled samples were pooled equally and desalted again with a solid-phase extraction cartridge. The desalted sample was dried before further LC/LC-MS/MS analysis.

### Extensive Tandem Mass Tag-Based Liquid Chromatography/Liquid Chromatography-Mass Spectrometry/Mass Spectrometry Analysis

The pooled TMT labeled peptides were fractionated by an offline basic pH RPLC with a XBridge C18 column (3.5 μm particle size, 4.6 mm × 25 cm, Waters; buffer A: 10 mM ammonium formate, pH 8.0; buffer B: 90% AcN, 10 mM ammonium formate, pH 8.0). The peptides were eluted in a 160 min gradient of 15–50% buffer B, and 160 fractions were collected every min and concatenated to 40 fractions. Each fraction was analyzed on a self-packed column (75 μm × 15 cm with 1.9 μm C18 resin) coupled with a Q Exactive HF Orbitrap MS (Thermo Fisher Scientific). The peptides were eluted at 0.25 μL/min flow rate with a 60 min gradient of 18–45% buffer B (buffer A: 0.2% formic acid, 5% DMSO; buffer B: buffer A plus 65% AcN). The mass spectrometer was operated in data-dependent mode with a MS1 scan in Orbitrap mass analyzer [450–1600 *m/z*; 60,000 resolution; 1 × 10^6^ automatic gain control (AGC) target; 50 ms maximum ion time] and 20 data-dependent MS2 scans (60,000 resolution, 1 × 10^5^ AGC target, 110 ms maximum ion time, 32% HCD normalized collision energy (NCE), 1.0 *m/z* isolation window, 0.2 *m/z* isolation offset, and 10 s dynamic exclusion).

### Identification and Quantification of Proteins

Protein identification and quantification were performed using the JUMP search engine (Wang et al., 2014). The human protein database was generated by combining downloaded Swiss-Prot, TrEMBL, and UCSC databases and removing redundancy (83,955 entries). The target-decoy database was used to estimate the false discovery rate (FDR) (Peng et al., 2003). Major search parameters included precursor mass tolerance (±20 ppm) and product ion mass tolerance (±15 ppm), full trypticity, two maximal missed cleavages, static modification for TMTpro tags (+304.20715 on Lys and N-termini) and carbamidomethylation (+57.02146 on Cys), dynamic modification for oxidation (+15.99491 on Met). The resulting PSMs were filtered by mass accuracy and then grouped by precursor ion charge state followed by the cutoffs of JUMP-based matching scores (Jscore and ΔJn) to reduce FDR below 1% for proteins. When the same peptide was derived from numerous homologous proteins, the peptide was matched to the protein with the top PSM number, according to the rule of parsimony. Protein quantification was performed using TMT reporter ion intensities by a built-in program of JUMP software suite as previously described (Niu et al., 2017).

### Differentially Expressed Proteins Thresholding

The differential expression (DE) analysis was performed following the previously reported method (Bai et al., 2020). Briefly, the ratios of all proteins between the replicates were modeled with a Gaussian distribution to evaluate standard deviation for *z* value analysis. The protein *p* values were calculated by using the moderated *t*-test (Phipson et al., 2016). The proteins with *p*-value < 0.05 and *z* > 2 were considered as DE proteins. All DE proteins are listed in Supplementary Table 1.

### Pathway Enrichment Analysis

All 601 DE proteins from the proteomics analysis, including both up and downregulated proteins, were considered for pathway enrichment analysis. Functional enrichment analysis was performed on the STRING database (version 11.5), where each DE protein was ranked by its log_2_fold change value. DE proteins were significantly enriched in a total of 67 pathways in the “biological processes” category. The downregulated proteins were enriched in a single pathway, “locomotory behavior (GO:0007626).” All the upregulated proteins were enriched in the remaining 66 pathways. All the significantly enriched pathways, GO-terms, enrichment score, pathway size, and the number of proteins mapped to each pathway are given in Supplementary Table 2.

### Immunohistochemistry of Post-mortem Brain Samples

We performed immunohistochemistry on post-mortem brain samples from AD and age-matched NS donors; their demographic and clinical information is listed in Table 5. The formalin-fixed paraffin-embedded human AD and NS tissues were sectioned at 5 μm and mounted onto glass microscope slides. The slides were placed on a 60°C hotplate for 30 min and moved through HistoChoice clearing aged (2 washes, 5 min each) to dewax the paraffin. Next, the sections were rehydrated by submerging the slides in the following solutions:100% ethanol (2 washes, 5 min each), 95% ethanol (1 wash, 5 min), 70% ethanol (1 wash, 5 min), 1×PBS (1 wash, 5 min). For the sections to be treated with BST2 and PDPN, antigen retrieval was performed by boiling the sections in a citrate buffer in the microwave for 3 min. The sections were then treated with a blocking buffer (10% normal goat serum and 0.4% Triton X-100 prepared in 1×PBS) for 1 h at room temperature in a humid chamber. Following blocking, they were treated with the primary antibodies and incubated overnight (16–20 h) at 4°C in a humid chamber. The following day, sections were washed with 1×PBS (3 washes, 10 min each) and treated with the secondary antibodies for 1 h at room temperature in a darkened humid chamber. To remove unbound secondary antibodies 3 10-min washes in 1×PBS was performed followed by a nuclear co-stain with DAPI (0.5 μg/mL prepared in PBS) for 1 min. The sections were then treated with TrueBlack Lipofuscin Autofluorescence Quencher (Biotium) for 1 min to reduce background fluorescence. Finally, sections were mounted with Fluoromount-G, and a 22 × 50 mm cover glass was placed onto the sections. Images were acquired on a Keyence BZ-X710 using a 60× oil objective. All antibodies used are listed in the Table 3. For quantification, 3 different tissue sections and 3 different ROI per section for each condition (AD and NS) were used. Unpaired *t*-tests were performed using the means from each tissue.

**TABLE 5:** Clinical characterization of post-mortem brain patient donors.

Sex	Age	A (Amyloid)	B (Braak)	C (CERAD)	Clinical diagnosis
Female	89	A1	B1	C1	Non-symptomatic
Female	95	A0	B0	C0	Non-symptomatic
Male	88	A2	B2	C2	Non-symptomatic
Male	92	A6	B6	C6	Alzheimer’s disease
Female	80	A5	B5	C5	Alzheimer’s disease
Female	92	A7	B7	C7	Alzheimer’s disease

## Data Availability Statement

The proteomic dataset generated during this study can be found on the MassIVE open-source platform https://massive.ucsd.edu/ProteoSAFe/dataset.jsp?task = 3b7ee 26c 66854e00ad77b3e3dc1d832d (ID: MSV000088799). The R source code used for analysis can be found on GitHub at https://github.com/JUMPSuite/JUMPq_python.

## Author Contributions

VF: conceptualization and project administration. DL: validation. DL, ZW, PP, MZ, MDS, and PF: formal analysis and investigation. DL, LB, and MLS: training and troubleshooting. VF, DL, and SL: visualization. VF and DL: writing–original draft. All authors: writing–review and editing. VF, SL, JP, and PC: supervision. VF, SL, JP, PF, and PC: funding acquisition. All authors contributed to the article and approved the submitted version.

## Conflict of Interest

NYSCF U.S. Patent Pending for CD49f. SL is a founder and sits on the SAB of AstronauTx Ltd. The remaining authors declare that the research was conducted in the absence of any commercial or financial relationships that could be construed as a potential conflict of interest.

## Publisher’s Note

All claims expressed in this article are solely those of the authors and do not necessarily represent those of their affiliated organizations, or those of the publisher, the editors and the reviewers. Any product that may be evaluated in this article, or claim that may be made by its manufacturer, is not guaranteed or endorsed by the publisher.

## Supplementary Material

Supplementary Table 1 List of DE proteins from whole proteome analysis.

Supplementary Table 2 List of biological processes identified through pathway enrichment analysis.
