# Supplementary material for: Proteomic Alterations and Novel Markers of Neurotoxic Reactive Astrocytes in Human Induced Pluripotent Stem Cell Models
Source: Front Mol Neurosci. 2022 May 3;15:870085. doi: 10.3389/fnmol.2022.870085 (PMC9113221; doi:10.3389/fnmol.2022.870085)
Supplement: Supplementary file 1 [file Data_Sheet_1.pdf]

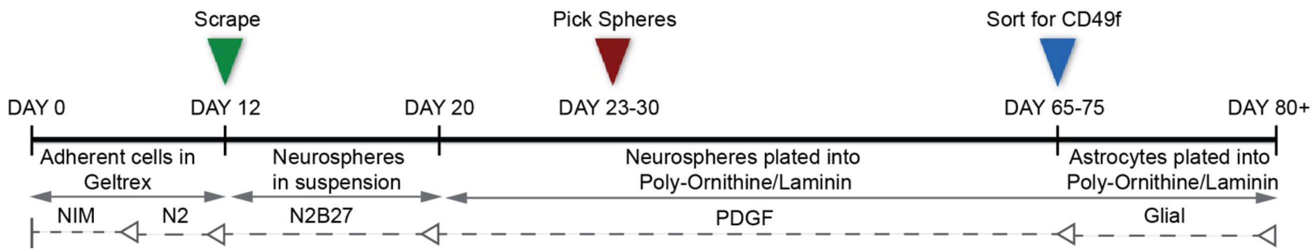

Neural Induction medium (NIM; d0 - d7)

Reagents	Conc
mTesr Custom	
PenStrep (100x)	1x
SB431542	10 µM
LDN193189	250nM
Retinoic acid	100nM

N2 medium (d8 - d11)

Reagents	Conc
DMEM/F12	
PenStrep (100x)	1x
2-Mercaptoethanol (1000x)	1x
MEM non-essential amino acids (NEAA)	1x
N2 supplement (100x)	1x
Retinoic acid	100nM
Smoothened agonist (SAG)	1µM

N2B27 medium (d12 - d19)

Reagents	Conc
N2 medium	
B27 supplement without VitA (50x)	1x
Insulin solution, human	25µg/mL

PDGF medium (d20 - sort)

Reagents	Conc
DMEM/F12	
PenStrep (100x)	1x
2-Mercaptoethanol (1000x)	1x
MEM non-essential amino acids (NEAA)	1x
N2 supplement (100x)	1x
B27 Supplement without VitA (50x)	1x
Insulin solution, human	25µg/mL
PDGFaa	10ng/mL
IGF-1	10ng/mL
HGF	5ng/mL
NT3	10ng/mL
T3	60ng/mL
Biotin	100ng/mL
cAMP	1µM

Glial medium (post sort)

Reagents	Conc
DMEM/F12	
PenStrep (100x)	1x
2-Mercaptoethanol (1000x)	1x
MEM non-essential amino acids (NEAA)	1x
N2 supplement (100x)	1x
B27 Supplement without VitA (50x)	1x
Insulin solution, human	25µg/mL
T3	60ng/mL
Biotin	100ng/mL
cAMP	1µM
HEPES	10mM

Figure S1. Differentiation and purification of hiPSC-derived astrocytes through a serum-free protocol. Schematic of astrocyte differentiation protocol from hiPSCs, including tables for media compositions and patterning agents.

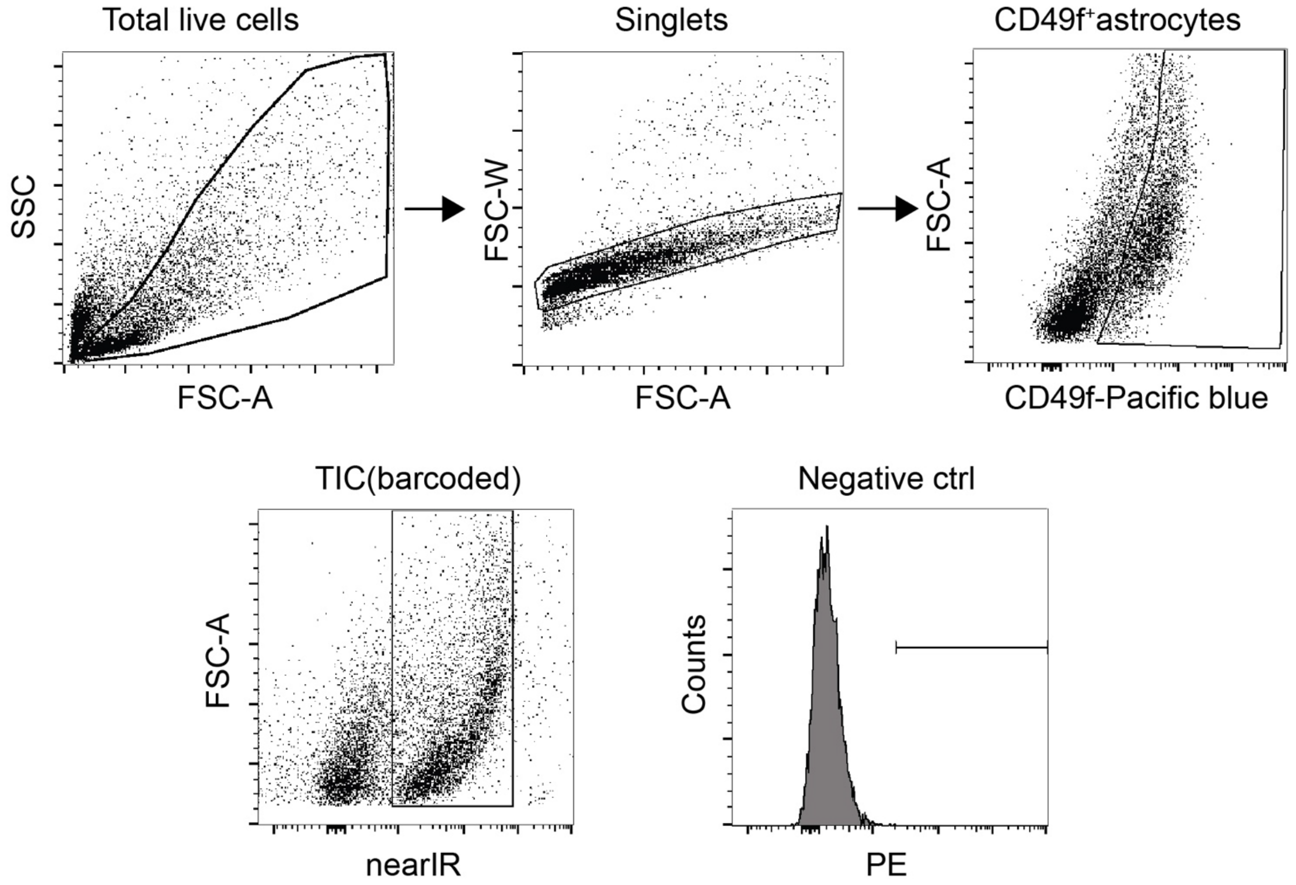

Figure S2. Gating strategy for flow cytometry screen. Representative gating strategy for CD49f^{+} astrocytes and barcoded TIC-induced reactive astrocytes for flow cytometry screen; a CD49f^{+} negative control is shown.

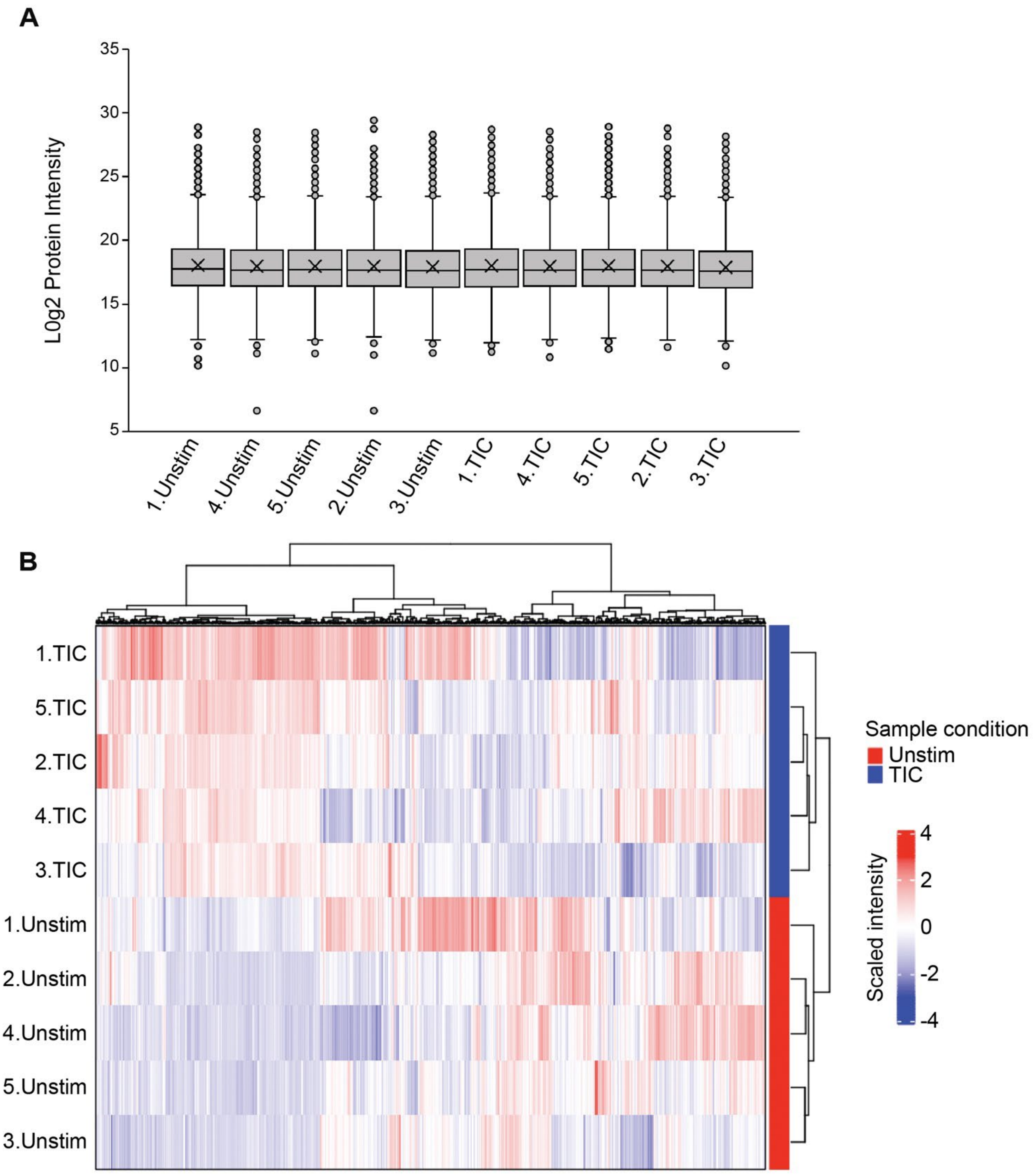

Figure S3. Quality control and hierarchical clustering for whole cell proteomics

(A) Box plot of protein intensities among different samples shows no obvious loading bias.

(B) Hierarchical clustering of top 10% most variable proteins shows that astrocytes from all hiPSC lines respond to TIC stimulation and cluster together, clearly separated from unstimulated astrocytes.

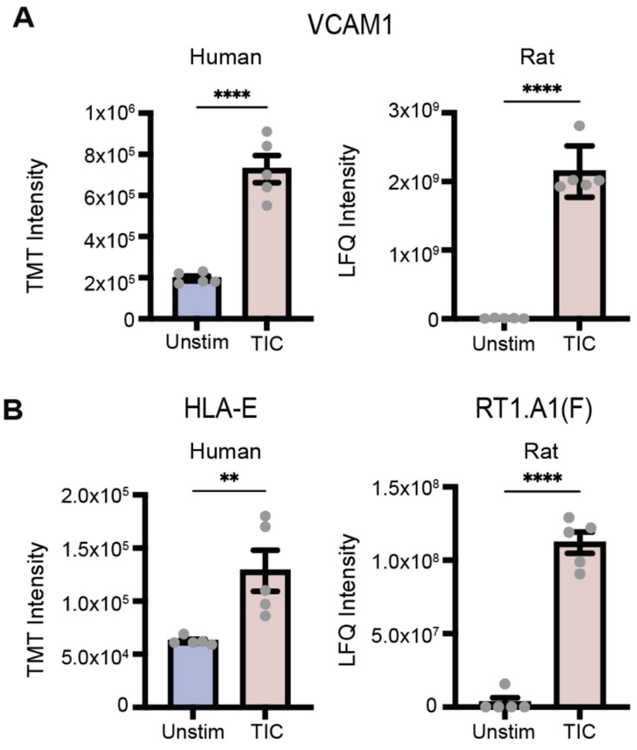

Figure S4. Human versus rodent protein expression

(A) Quantification of VCAM1 protein abundance in human versus rat, measured by TMT-based LC/LC-MS/MS and label-free quantitative LC-MS/MS, respectively.

(B) Quantification of human HLA-E protein abundance and rat RT1.A1(F) domain (HLA-E ortholog) protein abundance.

For both quantifications, individual dots are means of three technical replicates for each line; error bars represent standard error of means. *p*-value was calculated using a two-tailed unpaired t-test. **= p-value<0.01; ****=p-value<0.0001.

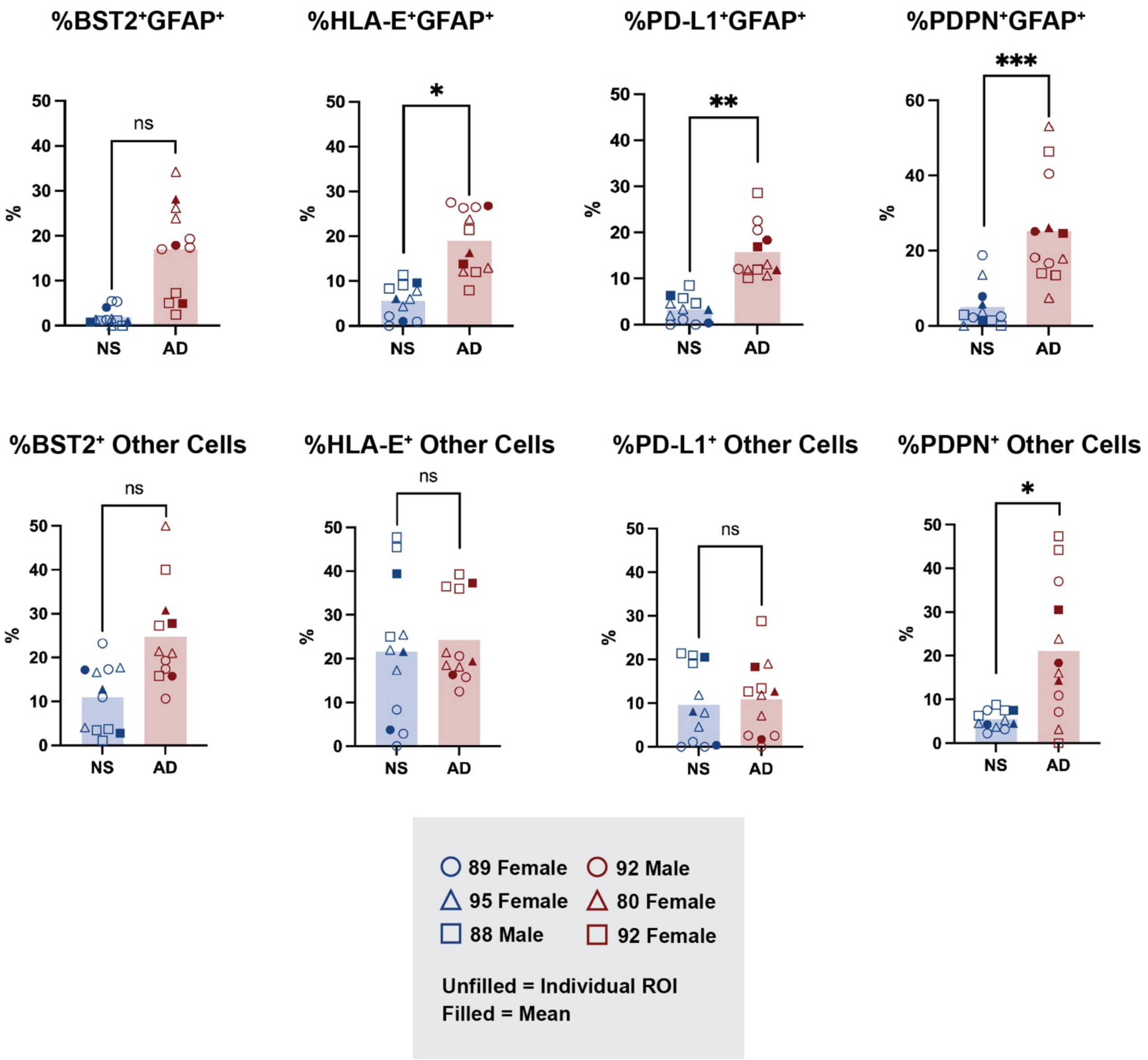

Figure S5. Quantification of TIC-reactive markers on human tissues.

Quantification of BST2, HLA-E, PD-L1 and PDPN from 3 different tissue sections from each condition (AD and NS). Plots show percentage of cells double positive for GFAP and marker over total DAPI cells (top) and percentage of marker positive/GFAP$^-$ cells (*i.e.* Other Cells in the CNS that are not astrocytes) over total DAPI cells. AD= Alzheimer's disease; NS= non symptomatic.
*= p-value<0.05; **= p-value<0.01; ***= p-value<0.001; ****=p-value<0.0001.

NYSCF The New York Stem Cell Foundation Research Institute

NYSCF ID: 051104-01-MR-040 Lot# E040-1E

Certificate of Analysis

Product Description	iPS Cell Line	
Publication(s) describing iPSC establishment	NA	
Parent cell line and cell type	051104-01-FB-001	Fibroblasts
Unique Parent Cell Line ID	10-005_1104	
Method of Reprogramming	mRNA	
Media	Freedom	
Cell Culture Matrix	Geltrex	
Passage method	Accutase	
Split ratio	1:10-1:20 every 5-7 days	
Reported Sex (Demographics)	Female	
Calculated Sex (DNA)	Female	

The following testing specifications have been met for the specified product lot:

Test Description	Test Method	Test Specification	Result
Post-Thaw Viable Cell Recovery	Cryotube thaw to single well of 12 well plate	>50% Confluency reached within 10 days	Pass
Sterility	SteriTEQ	Negative	Pass
Mycoplasma	Lonza MycoAlert Plus	Negative	Pass
Karyotype	Illumina CoreExome24	Normal Karyotype (No Autosomal CNVs >2.5 Mb)	Pass
Identity Match	Fluidigm SNPTrace Analysis	Match parent line	Pass
Pluripotency Expression Profile	Nanostring Pluripotency Scorecard Analysis	Express markers of pluripotency with absence of early differentiation markers	Pass
Differentiation Capacity	Nanostring 3 Germ Layer Scorecard Analysis	Ectoderm Analysis	0.02
Differentiation Capacity	Nanostring 3 Germ Layer Scorecard Analysis	Mesoderm Analysis	0.00
Differentiation Capacity	Nanostring 3 Germ Layer Scorecard Analysis	Endoderm Analysis	0.02

Notes

A negative score in the 3 germ layer analysis indicates a potentially reduced ability to differentiate into the corresponding germ layer. An asterisk indicates the line performed outside of the range of reference lines used in this analysis. For more information see Bock et al., 2011 (DOI: 10.1016/j.cell.2010.12.032).

☒Pass
☐Fail
☐Other:

Daniel Paull, PhD
Vice President, Stem Cell Technology Platforms
Date: 03/02/2020

Figure S6. Representative Certificate of analysis of hiPSC line. CoA for line 2, detailing quality control tests performed after reprogramming.
